# The influence of psychological ownership on pride in a Citizen Science project on wildlife ecology

**DOI:** 10.1371/journal.pone.0345321

**Published:** 2026-04-08

**Authors:** Hannah Greving, Till Bruckermann, Anke Schumann, Milena Stillfried, Konstantin Börner, Robert Hagen, Sophia E. Kimmig, Miriam Brandt, Joachim Kimmerle

**Affiliations:** 1 Institute of Education, Leibniz University Hannover, Hannover, Germany; 2 Knowledge Construction Lab, Leibniz-Institut für Wissensmedien, Tübingen, Germany; 3 Biology Education, IPN – Leibniz Institute for Science and Mathematics Education, Kiel, Germany; 4 Science Management, Leibniz Institute for Zoo and Wildlife Research, Berlin, Germany; 5 Department of Ecological Dynamics, Leibniz Institute for Zoo and Wildlife Research, Berlin, Germany; 6 Wildlife Research Unit, Agricultural Centre Baden-Württemberg, Aulendorf, Germany; 7 Leibniz Institute of Freshwater Ecology and Inland Fisheries (IGB), Berlin, Germany; 8 Department of Psychology, Eberhard Karls University of Tübingen, Germany; Public Library of Science, UNITED KINGDOM OF GREAT BRITAIN AND NORTHERN IRELAND

## Abstract

Voluntary engagement is crucial for committed participation in Citizen Science (CS) projects. So far, the CS literature has argued that psychological ownership (i.e., subjective feelings of owning or possessing an object or entity) facilitates engagement in CS projects and is beneficial for several outcomes, such as attitudes toward CS. This paper argues that, as ownership is a self-relevant experience and facilitates effort and engagement, it should increase self-focused outcomes, such as the self-conscious emotion of pride. This is highly relevant for the CS context due to its voluntary character. In turn, pride may have uplifting effects and may trigger more engagement. Therefore, the research presented here investigated the interrelations between psychological ownership and pride in five two-month long, two-wave longitudinal field studies of a CS project on urban wildlife ecology using cross-lagged panel analyses of the data of 508 participants. It was hypothesized that ownership predicts pride over time and not vice versa, as ownership increases engagement, which in turn would trigger pride. It was found that, across all field studies combined, ownership had indeed a positive, time-lagged influence on pride. Thus, when CS participants voluntarily engage in a CS project that feels like their own, they also subsequently feel proud, which can motivate further voluntary CS engagement. The implications for the CS context are discussed.

## Introduction

Voluntary engagement is crucial for societal functioning and essential for running environmental research, such as in case of Citizen Science (CS) projects [1]. These are scientific research projects in which volunteering citizens and professional scientists collaborate with each other [2], for instance, in carrying out wildlife monitoring [3]. CS projects have been described in models on collaborative knowledge construction that emphasize both, individual learning outcomes and the development of collective knowledge [4–5]. Because of the voluntary nature of CS projects and their contribution to knowledge development, recent discussion has been concerned with the concept of ownership. Whereas most literature focuses on the legal aspects of ownership for project outcomes [6–9], there are only few studies that concern the individual feeling of ownership (i.e., psychological ownership) within the project.

Such previous research that focused on feelings of ownership hardly made any reference to ownership as a psychological construct, but rather described it as creating ownership [10], sense of ownership [11], or sense of attachment [12]. Furthermore, the focus in research on ownership was on individual outcomes (e.g., sensing, feelings) and ways to create it [10,12]. Less is known about psychological ownership as a prerequisite. Those studies that investigated ownership as an antecedent manipulated it with a recall task [13] or by artificially varying the degree of involvement [14]. As a consequence, feelings of psychological ownership increased pro-environmental intentions and behavior [13] as well as positive attitudes toward and intentions to participate in CS projects [14].

Yet, these previous studies were experiments that did not focus on environmental or CS projects in the field. Moreover, they did not investigate self-relevant, psychological consequences of feelings of psychological ownership in the CS context. These consequences need to be examined more often [15], and they are relevant as voluntary engagement in CS allows citizens to express their values, grow personally, or get emotionally involved [16–17]. Such emotional involvement can happen in the form of feelings of pride that have received little attention in the CS context [18–19] but are known to have uplifting effects which is why they are important for the CS context. Therefore, the research presented here focuses on psychological ownership and pride in the context of CS projects in the field.

### Psychological ownership

Following the theory of psychological ownership, ownership is the subjective feeling of owning or possessing an object or entity [20–21]. It is a cognitive-affective state that represents a close relationship between an individual and an object or entity [20–22]. Individuals can feel ownership for concrete, physical objects as well as for abstract entities. For example, employees have been shown to experience ownership for the organization they worked for [23]. As a consequence, ownership increases experienced responsibility, risk-taking behavior, making sacrifices, as well as engagement and efforts, and has uplifting effects on positive evaluations of the objects and entities concerned [20–21].

These effects of ownership can be transferred to the CS context. Citizens may experience ownership for the CS project for which they volunteer. For example, participants of the *Neighborhood Nestwatch Program* experienced ownership for this CS project [24]. Citizens can also experience ownership for more concrete entities, such as particular places (e.g., the beach [25]) or certain parts of a CS project (e.g., collected data [14]). These feelings of ownership in turn can encourage further engagement [21,25,26]. Although feelings of psychological ownership may need some time to develop, individuals have also been shown to experience ownership spontaneously [14,22]. Therefore, experiencing psychological ownership may be important for the beginning of participation in CS projects. Previous research has shown that, besides feelings of ownership, individuals can also be proud of their participation in CS projects [18–19].

### Pride

Pride is a positive, self-conscious emotion and represents a positive evaluation of one’s behavior and achievements [27–30]. Following the theoretical model of self-conscious emotions, pride is elicited in situations, in which self-representations are activated and positive outcomes are attributed to specific events [31]. As a consequence, pride has positive, uplifting effects and motivates further behavior [32], even when effort and perseverance are necessary [33]. For example, research has shown that pride elicits pro-environmental behavioral intentions [34], is related to engagement in pro-environmental behavior [35–36], and increases voluntary activities [37].

Analogously, pride is also relevant for CS projects. When citizens engage in CS projects, they do so voluntarily, usually without receiving any incentive, and invest much time and effort into the project. Upon successful completion of their CS participation, it seems natural that they would feel proud of their behavior and achievements, lending value to their contribution. For example, some CS research has reported (anecdotal) evidence that citizens indeed took pride in contributing to the CS project [18,38,39]. Importantly, pride is triggered by specific events (e.g., the time and effort invested voluntarily into the CS project) and its positive outcomes (e.g., successful completion of the CS project). Therefore, feelings of pride may be more relevant at the end of CS projects. This rationale is also in line with the model of self-conscious emotions which postulates that specific feelings, such as ownership, trigger effort which in turn increases feelings of pride [29–31].

So far, the connection between ownership and pride has rarely been studied in the CS context [14]. In previous research, experimental studies investigated the role of involvement in CS projects for experiencing ownership [14]. In these studies, even the completion of few tasks typical for ecological CS projects increased ownership. Notably, the involvement in the tasks had indirect effects on feelings of pride via ownership. This is an important finding for the research that is presented in this manuscript. Therefore, we argue that the connection between ownership and pride should be especially present in existing CS projects in which all participants engage in all relevant tasks of the project.

### The current research

In sum, we argue that self-focused emotions are highly relevant in the CS context as CS participants do not receive any material or other incentive and voluntarily participate in the projects. Therefore, it is important to examine which feelings and experiences they gather from their participation and how these experiences predict each other. As ownership is a self-relevant experience, it should influence the self-conscious emotion of pride more strongly than broader outcomes like attitudes [40]. Following our theoretical considerations above, we assume that experiencing psychological ownership for a CS project at the beginning of the project increases time and effort invested during the course of the project. These efforts subsequently trigger feelings of pride at the end of the project. To test this assumption, time-lagged associations between ownership and pride need to be investigated, which are lacking so far. Therefore, the research presented here investigated the interrelations between psychological ownership and pride in five two-month long, two-wave longitudinal field studies of a CS project on urban wildlife ecology using cross-lagged panel analyses. We hypothesized that CS participants’ psychological ownership has a positive, time-lagged influence on pride.

## Method

The studies presented here were all part of a CS project that had two main aims: (1) Conducting CS research on wildlife ecology (i.e., within the biological sciences) and (2) evaluating what citizens learned from the CS project (i.e., within the science of CS [41]). The CS project and the corresponding field studies were all prepared, conducted, and analyzed by the authors of this manuscript. In particular, there were five rounds of the CS project that took place in October/November 2018, April/May 2019, October/November 2019, April/May 2020, and October/November 2020. During each of these rounds, one field study was conducted, meaning that in total five field studies were conducted. The CS project took place in a German metropolitan city and citizens of this city participated in the field studies (see below for participants). Importantly, each participant could only participate once in a field study.

The CS project dealt with urban wildlife ecology and aimed to assess the occurrence of wildlife in the metropolitan city. The participating citizens were equipped with camera traps (i.e., cameras that automatically take pictures of passing wildlife). The task of the participants was to install these cameras in their gardens. Besides this outdoor activity, the CS project used an Internet platform for all other activities. On this platform, the participants uploaded the photographs of the camera traps, identified the wildlife species in the photographs, validated other camera trap photographs, analyzed the data in several steps, and discussed the results and other issues with other citizens and scientists.

### Design and procedure

We used a longitudinal design with two measurement points. Participants filled out two questionnaires, one at the start (T1) and one at the end (T2) of each field study, which were approximately two months apart from each other. We started to assess the T1 questionnaire of the first field study on the 25th of September 2018 and stopped the assessment of the T2 questionnaire of the fifth and final field study on the 13th of December 2020. Before filling out the questionnaires, participants provided written informed consent. We measured demographic data only at T1, otherwise, both questionnaires were identical. They assessed psychological ownership and pride in addition to other measures not relevant to the present research question. The studies were conducted following APA ethical guidelines, and all participants had provided written informed consent regarding the completion of the questionnaires. The local ethics committee of the Leibniz-Institut für Wissensmedien approved the questionnaires (LEK 2018/062).

### Participants

We used public-relations campaigns to recruit citizens from the public who applied for participating in the CS project and were selected based on the location where they lived in the metropolitan city [42]. However, each citizen could only participate once in one of the field studies. Across all five field studies, in total 898 individual participants completed the T1 questionnaire, and of these participants 508 also completed the T2 questionnaire (278 female, 229 male, 1 non-binary; *M*_age_ = 53.10, *SD* = 12.03, range = 19–83). Thus, 390 participants dropped out of completing the questionnaires over time (see Table 1 for dropouts of each field study separately), but notably did not drop out of participation in the CS project. This dropout rate is comparable to other studies on CS projects [43]. Those participants who completed both questionnaires did not differ from those who only filled out the T1 questionnaire in gender, χ^2^(2) = 0.71, *p* = .703, education, *t*(896) = –1.11, *p* = .268, experienced ownership at T1, *t*(896) = 0.77, *p* = .442, or feelings of pride at T1, *t*(896) = 0.11, *p* = .912. Participants who completed both questionnaires were a bit older than those who only filled out the first questionnaire, *t*(896) = –2.12, *p* = .034. Thus, participants who continued their engagement with the project but dropped out from the questionnaire were not less or more educated, and they did not experience less or more ownership or feelings of pride. This was a highly educated sample, as most participants (55.3%) had a higher education or university degree, followed by 13.0% with a doctoral degree or postdoctoral lecture qualification, and 8.5% with a general qualification for university entrance. The other 23.2% had a lower educational background. The demographic composition of our sample is comparable to the composition of other large CS projects [44,45].

**Table 1:** Number of Participants of the T1 and the T2 questionnaire and the corresponding dropout rate.

Field Study	Number of participants of the T1 questionnaire	Number of participants of the T2 questionnaire	Dropouts (in %)
**Field Study 1**	n = 196	n = 125	n = 71 (36.2%)
**Field Study 2**	n = 180	n = 60	n = 120 (66.7%)
**Field Study 3**	n = 162	n = 118	n = 44 (27.2%)
**Field Study 4**	n = 178	n = 107	n = 71 (39.9%)
**Field Study 5**	n = 182	n = 98	n = 84 (46.2%)
**Field Study 1–5**	N = 898	N = 508	N = 390 (43.4%)

### Measures

We measured ownership and pride with three items each on 5-point rating scales ranging from 1 *(does not apply at all)* to 5 *(completely applies)*. All items of both scales as well as their Cronbach’s alpha scores for each measurement point and each field study are presented in Table 2. We note that there is some variability in the Cronbach’s alpha scores for ownership (α_T1_s = .74 –.81; α_T2_s = .81 –.86) and pride (α_T1_s = .62 –.79; α_T2_s = .63 –.84), which could affect the precision of the measurements. Still, the overall Cronbach’s alphas were all good (ownership: α_T1_ = .78, α_T2_ = .84; pride: α_T1_ = .72, α_T2_ = .78).

**Table 2:** Number of Items, Wordings of all Items, Cronbach’s Alphas of each Measurement Point of each Field Study, and Used References of the Measures for Psychological Ownership and Pride.

Measure	N items	Items	Cronbach’s alphas	References
**Ownership**	3 (RS)	1. The ‘Wildlife Researchers’ project feels like my own project. 2. I feel like I own the ‘Wildlife Researchers’ project. 3. The ‘Wildlife Researchers’ project feels like it is mine.	1: α_T1_ = .79, α_T2_ = .83 2: α_T1_ = .76, α_T2_ = .86 3: α_T1_ = .79, α_T2_ = .84 4: α_T1_ = .74, α_T2_ = .86 5: α_T1_ = .81, α_T2_ = .81 1–5: α_T1_ = .78, α_T2_ = .84	[20–22]
**Pride**	3 (RS)	When I think about my participation in the ‘Wildlife Researchers’ project, … 1. … I am proud of myself. 2. … I am very satisfied with myself. 3. … I feel confident.	1: α_T1_ = .62, α_T2_ = .63 2: α_T1_ = .72, α_T2_ = .82 3: α_T1_ = .79, α_T2_ = .82 4: α_T1_ = .75, α_T2_ = .81 5: α_T1_ = .71, α_T2_ = .84 1–5: α_T1_ = .72, α_T2_ = .78	[27–28]

Abbreviations: RS = rating scale ranging from 1 *(does not apply at all)* to 5 *(completely applies)*; 1 = Field Study 1, 2 = Field Study 2, 3 = Field Study 3, 4 = Field Study 4, 5 = Field Study 5, 1–5 = Field Studies 1–5, α_T1_ = Cronbach’s alpha at the first measurement point T1, α_T2_ = Cronbach’s alpha at the second measurement point T2.

### Data analysis

To test our hypothesis, we conducted cross-lagged panel analyses that tested cause-effect relationships [46] between the variables of the two measurement points. We specified a simple cross-lagged panel model between ownership and pride at T1 and T2, respectively. In this model, all four variables were manifest variables that we measured. Moreover, this was a saturated model as we added all possible paths to the model (i.e., two autocorrelative paths, two cross-lagged paths, one correlation each at T1 and T2). All analyses were conducted with SPSS Amos v22.0 [47].

## Results

In the path model, we tested the autocorrelative paths of the variables, the two cross-lagged paths between them, and added the correlation between both variables at T1 and at T2, respectively. Therefore, this was a saturated model with fixed χ^2^ = 0, NFI = 1, CFI = 1, and RMSEA = 0 in all analyses. We tested this model in each of the five field studies separately as well as across all studies.

**Fig 1:**
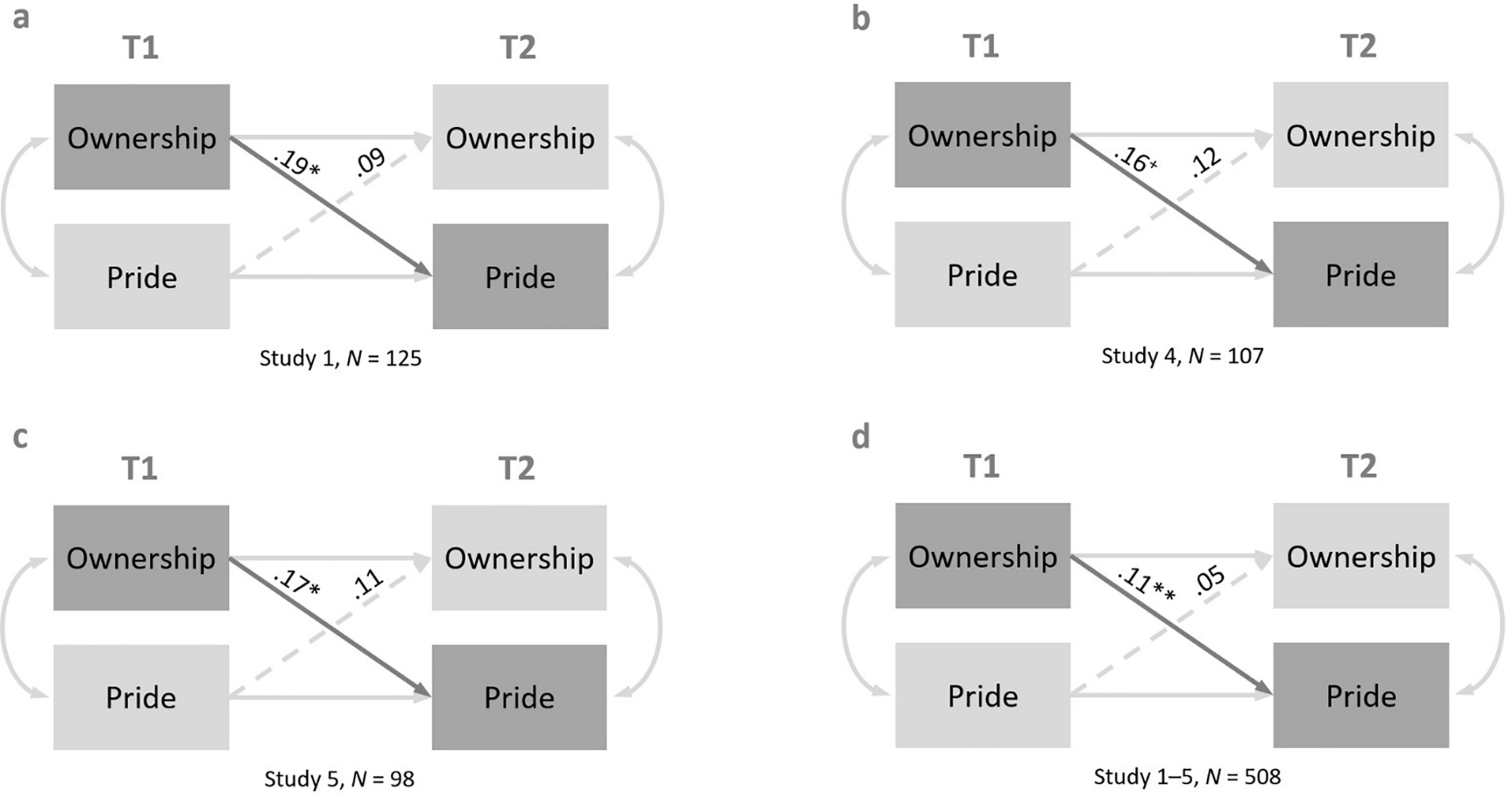
Path Models for Ownership and Pride at T1 and T2 with Autocorrelative and Cross-lagged Paths and Added Correlations for Field Study 1 (*N* = 125; a, top left), Field Study 4 (*N* = 107; b, top right), Field Study 5 (*N* = 98; c, bottom left), and across all five Field Studies (*N* = 508; d, bottom right). For all path models: Solid lines represent significant paths with *p* < .10, dashed lines represent non-significant paths with *p* > .10; **p < .01, * *p* < .05, and ^+^*p* < .10; NFI (Normed Fit Index) = 1, CFI (Comparative Fit Index) = 1, RMSEA (Root Mean Square Error of Approximation) = 0; for all cross-lagged paths of Field Studies 2 and 3, *p*s > .10.

In each study and across all studies, the variables were highly autocorrelated, all *p*s < .007. All test statistics of the cross-lagged paths are presented in Table 3. In Study 1, there was a positive relationship between ownership at T1 and pride at T2, but not between pride at T1 and ownership at T2 (see Fig 1, a, top left). In Studies 2 and 3, there were no significant cross-lagged paths. In Studies 4 (see Fig 1, b, top right) and 5 (see Fig 1, c, bottom left), there were again positive relationships between ownership at T1 and pride at T2, but not vice versa. Finally, across all studies (see Fig 1, d, bottom right), the path between ownership at T1 and pride at T2 was also significant (β = 0.11, *b* = 0.13, *SE* = 0.05, *p* = .005, 95% CI_B_ [0.038; 0.226]), but not the reverse path. Thus, overall, the results indicated that ownership had a positive, time-lagged influence on pride, which supported our hypothesis.

**Table 3:** Test Statistics with the standardized coefficient β, the coefficient *B*, the Standard Error (*SE*) of *B*, the *p*-value of *B*, and the 95% Confidence Interval around *B* of the Cross-Lagged Paths between Ownership and Pride for each Field Study and across all five Field Studies.

Field Study	Cross-lagged paths	Test statistics (β, *B* *[SE*], *p*, 95% CI_B_)
**1**	Ownership T1 – Pride T2	0.19, 0.17 (0.07), *p* = .021, [0.031; 0.309]
	Pride T1 – Ownership T2	0.09, 0.12 (0.10), *p* = .229, [–0.078; 0.318]
**2**	Ownership T1 – Pride T2	0.13, 0.15 (0.15), *p* = .313, [–0.150; 0.450]
	Pride T1 – Ownership T2	–0.17, –0.16 (0.11), *p* = .127, [–0.380; 0.060]
**3**	Ownership T1 – Pride T2	0.002, 0.002 (0.11), *p* = .984, [–0.216; 0.220]
	Pride T1 – Ownership T2	0.12, 0.10 (0.07), *p* = .163, [–0.039; 0.239]
**4**	Ownership T1 – Pride T2	0.16, 0.19 (0.11), *p* = .075, [–0.028; 0.408]
	Pride T1 – Ownership T2	0.12, 0.12 (0.08), *p* = .137, [–0.039; 0.279]
**5**	Ownership T1 – Pride T2	0.17, 0.21 (0.11), *p* = .043, [0.004; 0.420]
	Pride T1 – Ownership T2	0.11, 0.11 (0.08), *p* = .203, [–0.058; 0.268]
**1–5**	Ownership T1 – Pride T2	0.11, 0.13 (0.05), *p* = .005, [0.038; 0.226]
	Pride T1 – Ownership T2	0.05, 0.04 (0.04), *p* = .229, [–0.029; 0.117]

Abbreviations: 1 = Field Study 1, 2 = Field Study 2, 3 = Field Study 3, 4 = Field Study 4, 5 = Field Study 5, 1–5 = Field Studies 1–5, Ownership T1 – Pride T2 = relation between ownership at the first measurement point T1 and pride at the second measurement point T2, Pride T1 – Ownership T2 = relation between pride at the first measurement point T1 and ownership at the second measurement point T2.

## Discussion

The results of our studies demonstrated a positive, time-lagged influence of ownership on pride. The more participants felt ownership at the beginning of the CS project, the prouder they were of their participation at the end of the project. This was possibly the case because both feelings are related to the self and ownership increases engagement that triggers feelings of pride. This finding is in line with the theoretical model of self-conscious emotions [31] as it shows that specific events that are attributed to unstable self-relevant factors elicit pride. The finding also extends theories about the link between ownership of material products and pride [48] to more abstract entities, such as CS projects.

To the best of our knowledge, these are the first results in the CS context that have solely focused on self-relevant variables and how they influence each other. While in previous research psychological ownership has been mostly related to broader outcomes of CS, such as attitudes toward CS [40] or intentions to participate [14], the present results hint to the fact that ownership may especially affect other self-focused outcomes, such as pride. This is important, as CS projects can only be conducted with the voluntary help of citizens. It is reasonable that participants, who invested their time and resources into the project, take away something from their CS participation for themselves, especially an uplifting feeling, such as pride. This finding is relevant for CS practitioners, because with some feelings of ownership at the start of a project, participants feel proud of their participation, which is a pleasant feeling in itself, but which may also motivate further participation in CS projects [32–33]. Notably, we would like to acknowledge that ownership and pride were assessed in a larger battery of measures that yielded several different results [40,49,50]. The findings presented here represent one finding from five case studies of an ecological CS project. Further CS research needs to strengthen the link between ownership and pride. Still, we believe that self-relevant emotions and experiences as well as psychological phenomena [15] should receive more attention in the CS context as CS mostly depends on voluntary actions without giving any material or other incentives.

There are several strengths and limitations of our research that need to be discussed. A strength is that we conducted five field studies with a sample that was highly representative of CS projects concerned with monitoring wildlife. We used a longitudinal approach that has only rarely been used before in CS research. The cross-lagged panel analyses allowed us to test for cause-effect relationships [46], and the questionnaires included valid ownership and pride measures.

First, a limitation of our research is that we found the influence of ownership in most but not in all field studies when analyzed separately, such as in Field Studies 2 and 3. Possibly, such differing results could be due to differences in demographics or different procedures in project administration. Still, the two field studies were conducted during different seasons of the year and also differed in their drop-out rate. Moreover, the result across all studies might make up for this limitation. We also cannot conclude whether effort is the mechanism behind the effect of ownership on pride. Yet, this assumed mechanism is reasonable following theoretical models on ownership and pride [20,21,29–31]. Therefore, future research needs to investigate in more detail whether this assumption holds.

Second, the dropouts of the field studies varied between 27.2% and 66.7%. This variation may affect the interpretation of the results. Still, the comparison between the dropouts and the non-dropouts showed no consistent differences between these subsamples. Moreover, the sample was comprised of mostly highly educated citizens. It can be discussed whether the current findings also hold for less educated citizens with a more diverse background. Still, such a high educational background is a common characteristic of CS projects on wildlife ecology [44,45]. Therefore, further research may examine in more detail whether the effects also hold for other samples. We also only investigated the self-relevant outcomes ownership and pride and did not examine how these outcomes were related to other CS outcomes (insights on ownership, pride, and other affective and cognitive CS outcomes are documented elsewhere [14,40,49–51]). Such relationships could also be investigated in future research.

Finally, we need to discuss the use of cross-lagged panel analyses. Experiments may be seen as gold standard for testing cause-effect relationships. One the one hand, cross-lagged panel analyses may not be as good as experiments because they do not manipulate variables and test their subsequent effects. On the other hand, especially in field studies, the manipulation of variables is often not possible and, in such cases, cross-lagged panel analyses can be used similarly well for testing cause-effect relationships [46]. We could also replicate our findings in several field studies and across all studies. In our analyses, we used saturated models, which means that the model fit indices are difficult to interpret and that the results also need to be interpreted with caution. But for such path models with small degrees of freedom, as we used them, we had a sufficiently large sample, when doing the analysis across all five field studies. Therefore, the results of our analyses can be interpreted with reasonable reliability.

All in all, self-relevant experiences like psychological ownership and pride need to be considered when investigating voluntary engagement, such as participation in CS. Our research presented evidence that psychological ownership has a positive influence on pride over time in an urban wildlife CS project. Thus, our research demonstrated that, when CS participants voluntarily engage in a CS project that feels like their own, they also feel proud at the end of their participation. Feelings of pride can then motivate further voluntary behavior, such as engaging again in CS projects.

## Supporting information

S1 Data Data sets for Field Study 1–5. (SAV)
